# Under the surface of teacher occupational wellness and effectiveness in higher education: a look into the mediator roles of work passion and emotion regulation via SEM analysis

**DOI:** 10.1186/s40359-024-01656-2

**Published:** 2024-03-20

**Authors:** Xiumin Yu, Tongxiu Ying

**Affiliations:** Hebei Normal University of Science &Technology, Qinhuangdao, 066600 China

**Keywords:** Occupational wellness, Effectiveness, Work passion, Emotion regulation, SEM analysis, Teaching in higher education

## Abstract

Teacher occupational wellness and effectiveness are crucial aspects of a teacher's capacity to contribute to the advancement of excellence in education. Nevertheless, there is a dearth of considerable studies regarding the interconnections between work passion and emotion regulation in higher education. This study developed a model to demonstrate the interplay between the above-mentioned constructs to fill this research gap. To gather this information, the required scales were sent to 401 different university professors. Based on the findings of Structural Equation Modelling (SEM) and Confirmatory Factor Analysis (CFA), it is suggested that work passion and emotion regulation have the potential to enhance teacher occupational wellness and effectiveness in higher education. In the end, implications and directions for the future were presented to educators and researchers who are enthused about the potential of work passion, emotion regulation, and self-compassion for improving instructive practices.

## Introduction

Educating in higher education is intrinsically complex due to the multitude of external and internal variables involved. The outcome is contingent upon the self-perception of language teachers and the instructional techniques they use with their students. Typically, educators devise many teaching methods, influenced by the interplay of their individual and institutional dimensions [1, 2]. Educators have a crucial function in the educational system. The significance of university teachers in molding the student intellects should never be undervalued. Furthermore, it is evident that some professors possess a greater ability to exert influence on students compared to their peers.

In the course of this inquiry with educators, the first idea that is taken into consideration is work passion (WP). The notion of WP has garnered increased attention in the twenty-first century, as evidenced by the proliferation of research that highlights its advantageous consequences and how organizations can profit from employing an impassioned staff [3]. WP is a disposition toward action or endeavor that individuals esteem highly, find enjoyable, and devote a substantial amount of time and effort to [4]. Passion increases well-being, motivates individuals, and imparts significance to their existence. WP among university teachers may also lead to teacher effectiveness (TE). The efficacy of education is contingent upon the efficacy of instructors [5]. In such a case, it becomes essential to conceptualize the meaning of TE. The National Comprehensive Centre for Teacher Quality has developed a concise definition of TE, consisting of five characteristics [6]. Firstly, an effective teacher sets high expectations for all students and supports them in achieving their goals. Secondly, an effective teacher positively influences students' academic, social, and attitudinal outcomes. Thirdly, an effective teacher utilizes a variety of resources to plan and structure learning opportunities. Fourthly, an effective teacher promotes diversity and civic-mindedness within schools. Lastly, an effective teacher collaborates with colleagues, parents, and school administration.

Efficiency and production are two factors that teachers may use to determine whether or not they will be successful in their careers. In their definition of teacher effectiveness, [7] outlined the interplay between internal factors (such as instructors' motivation, beliefs, and dispositions) and external factors (such as students' cultural, social, and educational backgrounds) that influence students' final results. Given that discoveries about TE have significant implications for education policy and reform, the correlates of TE become a matter of utmost importance. For teachers to be able to teach in a manner that is inventive, motivating, and meaningful, they need to be in excellent emotional and mental health. In the course of their trip through the world of education, teachers could feel a wide range of emotions. These events and feelings have a significant influence on their ability to succeed as well as on the achievement of their students. It is believed that teachers who effectively regulate their emotions throughout their work are more accomplished [8]. In accordance with the definition provided by [9], the concept of teacher emotion regulation (ER) refers to the capacity of a professor to control and administer their own emotional experiences and expressions. Teacher ER gives educators the ability to control the intensity and length of their emotional contact in the context of their professional work [10]. It will be substantially more difficult for teachers to show their efficacy as a result of these changes. It is necessary to do further study on the subject of ER since it is still in its infancy, especially in the realm of higher education [11].

Occupational wellness **(**OW) involves maintaining a healthy balance between work and leisure activities to promote well-being, personal fulfillment, and financial success. The OW component is influenced by nurturing. The dimension recognizes personal satisfaction and improvement in an individual's life via employment. In a study by [12], four interconnected resources that contributed to the teachers' OW are highlighted: Psychological, social, human, and health capitals. Research to depict OW in the realm of language teaching in particular higher education is scarce. To fill this lacuna, the current research has constructed a mediation model to investigate the potential transmission of OW and TE to WP and ER within the context of higher education. This investigation offers an opportunity to enhance the understanding by examining the underlined connections. The data collected have sparked a discussion and created possibilities for subsequent studies.

## Literature review

As a motivational process, WP helps teachers tackle different activities successfully. WP manifests itself in their propensity to engage in physically demanding tasks, which they eventually come to consider as fundamental to who they are [13]. As per the dichotomous paradigm for passion established by [14], passion may be categorized into two distinct forms: harmonious and obsessive. Harmonious passion is the result of an individual's autonomous engagement in an activity and its assimilation into their character. Harmonious passion refers to the deliberate acceptance of behaviors that are deemed important and meaningful, promoting a feeling of unity with one's whole being. Obsessive passion occurs when an individual internalizes an activity to the point that it becomes incorporated into their psyche, resulting in a sense of control. This preoccupation is often initiated by internal pressures and/or external factors linked to self-esteem or societal validation, or by the excessive level of enthusiasm generated by the engaged activities [15]. An increasing number of scholars have directed their attention towards investigating the impacts of passion in an academic setting. These researchers have established connections between passion and various academic outcomes, including students' academic achievement, intentional effort, perseverance, goal-oriented thinking, resilience in learning, and overall well-being [13, 16] Research has indicated that an increase in a learner's passion correlates positively with their propensity to maintain concentration on enhancing their self-competence [17, 18].

Considering TE, the educational frameworks developed by [6] and [19] are extensively used in educational environments to ascertain the efficacy of instructors in the modern day [18] 's procedure defines the four categories that are used to assess teachers' effectiveness: organizing and preparing, the setting of the classroom, teaching, and job responsibilities. Similarly, [6] presented his framework as ten inquiries that represent the correct order for effective educational design. These inquiries include setting learning goals, providing opportunities for learners to apply what they have learned and deepen their comprehension, facilitating student interaction with new knowledge, interacting with learners in the educational process, fostering productive relationships between learners and educators, implementing successful instructional strategies, communicating high expectations for learners, and employing effective, standards-based formative and summative assessment methods that utilize multiple indicators of student competency. According to [20], TE refers to their capacity to teach successfully in the classroom. Moreover, [21, 22] argued that efficiency for instruction is a complex concept that is difficult to define due to its intangible nature. The present research is grounded on self-efficacy theory [23] and productivity theory [24]. Self-efficacy, as defined by Bandura, refers to an individual's belief in their ability to effectively affect their activities, leading to successful outcomes.

ER, the last construct in this research, refers to a broad framework that involves psychological, cognitive, and biological elements. It is used to effectively change conditions of emotion [25]. The concept of ER is not static, but rather a fluid process that impacts and motivates individuals' emotional experiences and expressions [26]. ER affects not only the initiation, but also the length and delay of emotional reactions, as well as cognitive, emotional, and bodily functions [27]. Creating a favorable emotional atmosphere enables instructors to better regulate not only their own emotions but also the emotions of their students. To elucidate the construct of teacher ER, [28] devised a model consisting of six dimensions. These dimensions are Situation selection, Situation alteration, Attention deployment, Reappraisal, Suppression, and Seeking social support. The first three dimensions of the model were formulated based on Gross' process model of ER [29, 30]. The reappraisal and suppression aspects were developed based on the results of [26]. In their pursuit of social support, they used the research conducted by [2] and [31]. This model was applied in the present research. One study that looked at the link between burnout, classroom norms about emotional expression, and ER methods was the one conducted by [32] among teachers. The findings of [11] in higher education evidenced that university instructors who possess a comprehensive understanding of productive immunity and ER exhibit more resilience and autonomy. More precisely, ER provides university professors with the means to effectively address the challenges and difficult circumstances that arise in their careers. Moreover, the study by [33] uncovered that the emotional regulation, reflective teaching, self-efficacy, and identity of language instructors could be important factors influencing their psychological well-being. This study emphasizes the need to include reflective practices, emotional management skills, self-efficacy beliefs, and identity reconstruction within teacher training programmers' curricula.

## Methodology

### Research questions and aims

Researchers have not yet examined the connections between OW, TE, WP, and ER in terms of their effectiveness in helping EFL teachers in higher education. Given the scarcity of research in this particular domain and the criticality of the enumerated elements in higher education, the objective of this investigation was to assess the effects of WP and ER on OW and TE among EFL instructors. The results of this research could potentially yield beneficial consequences for educators and learners, encompassing both theoretical and practical domains. In consideration of these perspectives, the subsequent areas of inquiry are suggested:

- **RQ1:** Are work passion and emotion regulation for EFL university teachers indicative of their occupational wellness?
- **RQ2:** Are work passion and emotion regulation for EFL university teachers indicative of their effectiveness?

### Context and participants

There was a total of 401 individuals who took part in this research; among the language teachers, 250 were men and 151 were women. In China, they were teaching in higher education. Their ages range from twenty-nine to fifty-one, and their years of teaching experience range from a year to twenty-five. Among the participants, 401 had a PhD degree, while the remaining individuals had a master's degree in Applied Linguistics.

### Research tools and procedures

#### The occupational well-being scale

The 12-item Occupational Well-Being Scale (OWS) developed by [34] was used to assess teachers' well-being. This measure is designed to assess the overall health and happiness of educators. The participants were asked to rate how much they felt anxious, content, depressed, or enthusiastic concerning their occupation in the previous week. Anxiety is defined as a state of tension-ridden unease, or worry; contentment is defined as a state of being at ease, happy, or relaxed; and despair is defined as a state of sad, depression, or drained. From 1 (never) to 6 (always), there were a total of six possible answers. Based on Table 1, dependability in this investigation was good.

**Table 1:** The reports of Cronbach's Alpha

		N	Cronbach's Alpha
Occupational Wellness	total	12	0.741
Teacher Effectiveness	Preparation and Planning for Teaching	6	0.872
	Classroom Management	7	0.862
	Knowledge of Subject Matter and its Delivery	5	0.879
	Teacher Characteristics	3	0.806
	Interpersonal Relations	4	0.881
	total	25	0.938
Work Passion	Harmonious Passion	7	0.762
	Obsessive Passion	7	0.720
	total	14	0.739
Emotion Regulation	Situation Selection	5	0.878
	Situation Modification	5	0.924
	Attention Deployment	4	0.882
	Reappraisal	5	0.892
	Suppression	4	0.814
	Seeking Social Support	4	0.788
	total	27	0.862

#### The teacher effectiveness scale

The evaluation of TE was carried out with the assistance of the scale that was manufactured and verified by [35]. On this scale, which is comprised of 25 items that are separated into five sub-sections, some of the characteristics that are included are preparation and planning for teaching, classroom management, mastery of subject matter and its delivery, teacher attributes, and interpersonal interactions. In all, five different replies could be given, ranging from 1 (never) to 5 (always). In line with Cronbach's alpha, which varied from 0.806 to 0.881, the ABS dependability in this investigation was good.

#### The work passion scale

The work passion scale (WPS) by [3] was used for evaluating WP. The scale has 14 questions, with 7 items measuring harmonious passion and the remaining 7 items evaluating obsessive passion. The answer scale spanned from 1 ("Strongly disagree") to 7 ("Strongly agree"). According to Table 1, the internal consistency of WPS is acceptable.

#### The language teacher emotion regulation inventory

The language teacher's emotion regulation inventory (LTERI) evaluated participants' ER techniques [28] developed this scale, which includes 27 questions and six sub-factors: scenario selection, situation alteration, attention deployment, reappraisal, suppression, and seeking social support. The LTERI questions were designed to be answered on a five-point Likert scale, with 1 indicating "never" and 5 representing "always". Cronbach's alpha scores ranged from 0.788 to 0.924, indicating excellent reliability for the LTERI in this study.

A web-based platform was created to facilitate the data collection process for this research project over a period of five months in 2023. The objectives of this four-part survey are to evaluate OW, TE, WP, and ER. To Analyze the data, the data was screened to check its normality using the Kolmogorov-Smirnov (K-S) test. Once it was determined that the data followed a normal distribution, parametric procedures were suggested for data analysis. The analysis was performed using CFA and SEM with Linear Structural Relations (LISREL) 8.80. As stated by [36], SEM is a reliable multivariate process that is used to verify the suggested structural theory using a confirmatory hypothesis-taking strategy. The measurement model and the structural model are the two components of a SEM model [37]. The links between the latent variables and the observable variables are investigated using the measurement model. The associations between the latent variables are measured using the structural model. It is recommended to use CFA to assess all latent variables before testing a structural model [38].

## Results

This section presents a concise overview of the data analysis, along with an elaborate explanation of every element of the report. Firstly, the gathered data underwent the K-S test to identify any patterns in the recurring presentations.

Table 2 shows that the significance values of all the instruments and their components were more than 0.05. This suggests that the findings follow a normal distribution, which justifies the use of parametric approaches for analyzing the data.

**Table 2:** The results of the K-S test

	Kolmogorov-Smirnov Z	Asymp. Sig. (2-tailed)
Occupational Wellness	0.878	0.424
Preparation and Planning for Teaching	0.589	0.879
Classroom Management	0.846	0.472
Knowledge of Subject Matter and its Delivery	1.135	0.152
Teacher Characteristics	0.889	0.408
Interpersonal Relations	0.724	0.671
Teacher Effectiveness	0.575	0.895
Harmonious Passion	0.940	0.340
Obsessive Passion	0.893	0.402
Work Passion	1.258	0.085
Situation Selection	1.350	0.052
Situation Modification	1.117	0.165
Attention Deployment	1.252	0.129
Reappraisal	1.307	0.066
Suppression	1.312	0.064
Seeking Social Support	0.926	0.358
Emotion Regulation	0.865	0.443

After that, the relationships between OW, TE, WP, and ER were investigated using structural equation modeling and a causal analytic framework. A statistical program termed LISREL 8.80 was employed to conduct these experiments. The chi-squared magnitude, RMSEA, CFI, GFI, and NFI were among the metrics used to assess the reliability of the model's predictions in relation to the actual data.

**Fig. 1:**
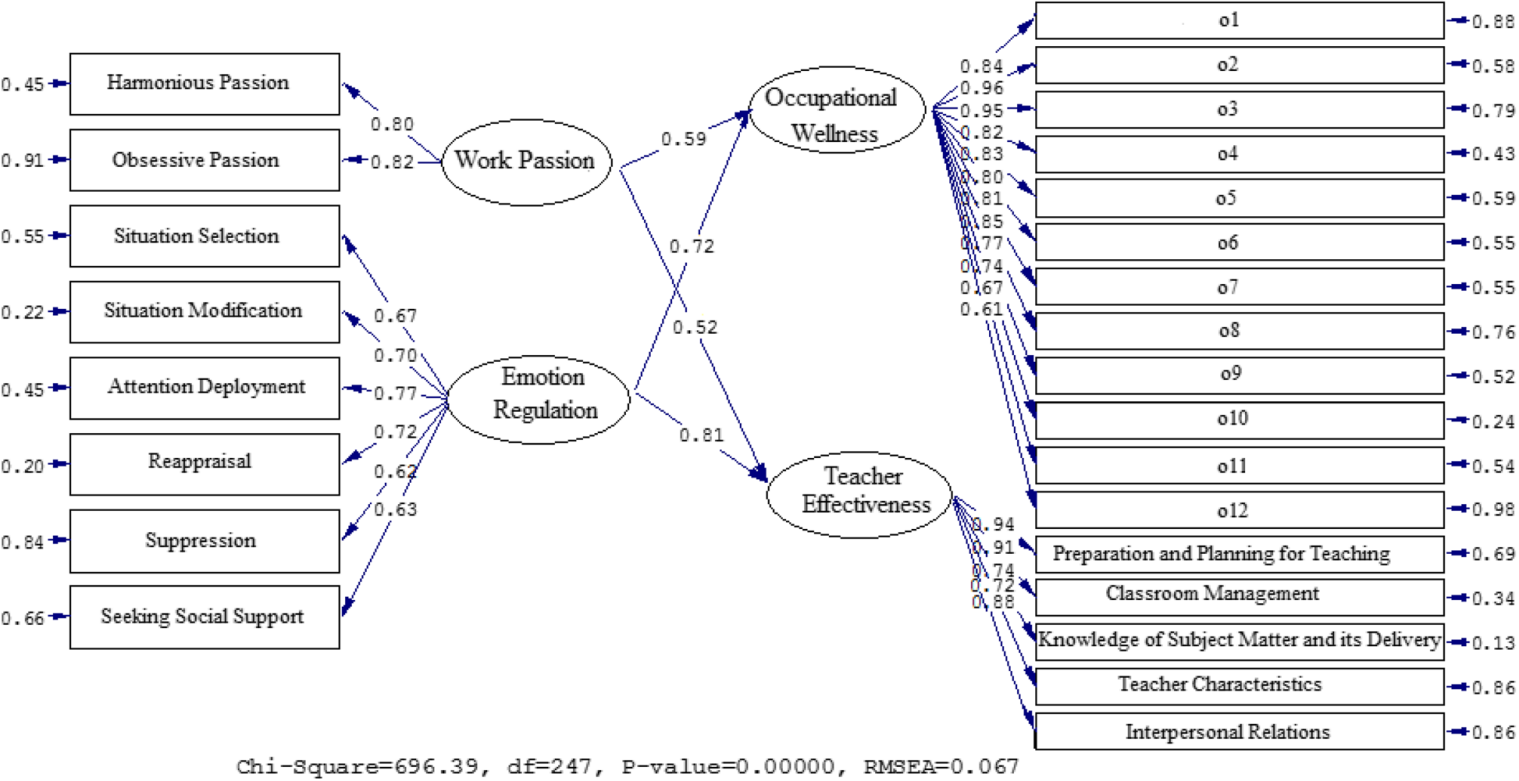
The Path Coefficient Values for the Connection among OW, TE, WP, and ER (Model 1)

**Fig. 2:**
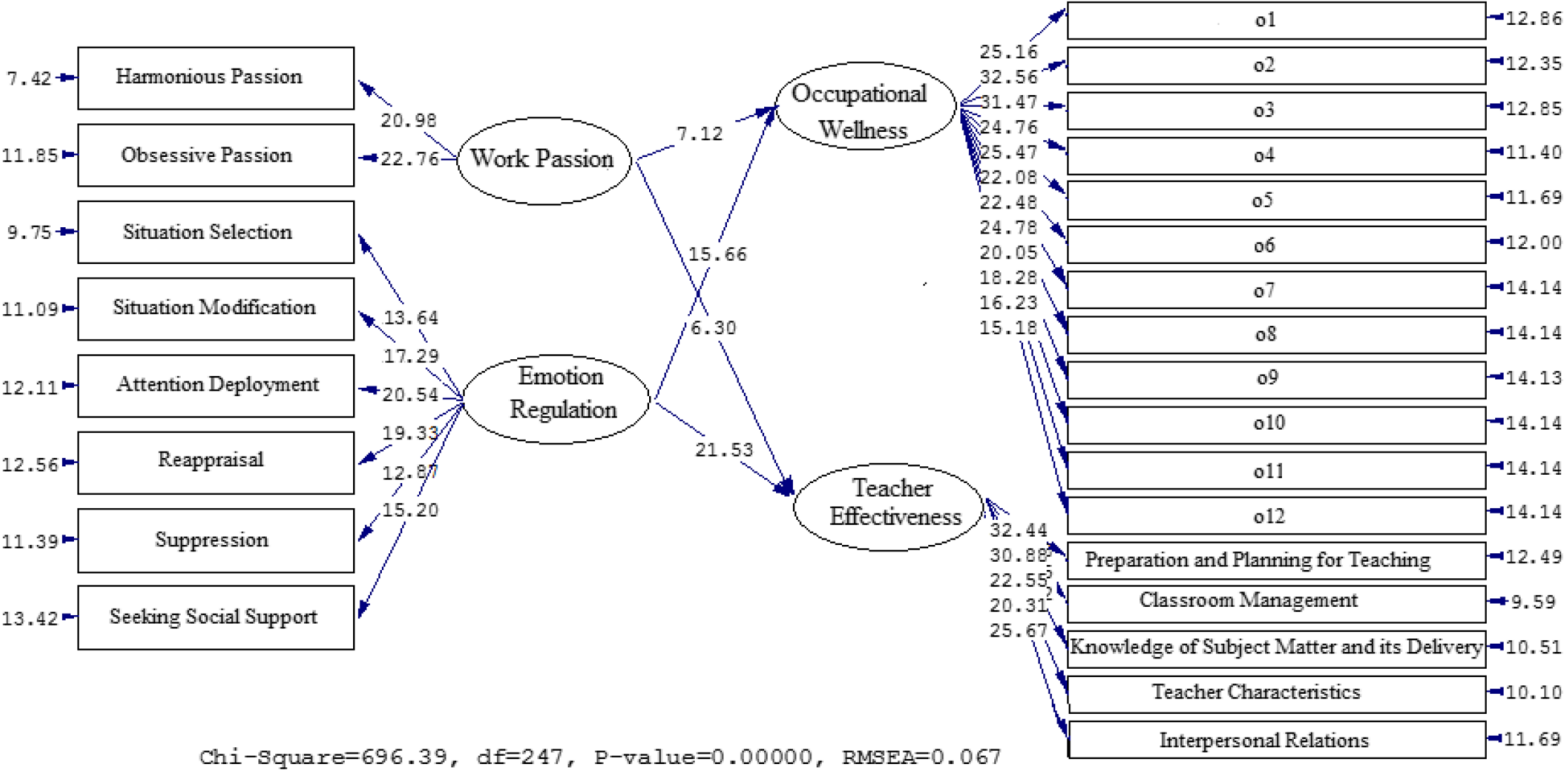
Path Coefficients with T Significance Values (Model 1)

Figures 1 and 2 vividly illustrate the relationship between the factors. Table 3 presents standardized estimates and t-values to analyze the impact of OW, TE, WP, and ER. WP has a significant positive effect on occupational wellness with a beta coefficient of 0.59 and a t-value of 7.12. Similarly, WP also has a significant positive effect on TE with a beta coefficient of 0.52 and a t-value of 6.30. Additionally, ER has a significant positive effect on OW with a beta coefficient of 0.72 and a t-value of 15.66. ER has a positive impact on TE (β = 0.81, t = 21.53).

**Table 3:** A synopsis of the results of model 1

**Paths**			**Path coefficient**	**T Statistics**	**Test results**
Work Passion	→	Occupational Wellness	0.59	7.12	Supported
Work Passion	→	Teacher Effectiveness	0.52	6.30	Supported
Emotion Regulation	→	Occupational Wellness	0.72	15.66	Supported
Emotion Regulation	→	Teacher Effectiveness	0.81	21.53	Supported

**Fig. 3:**
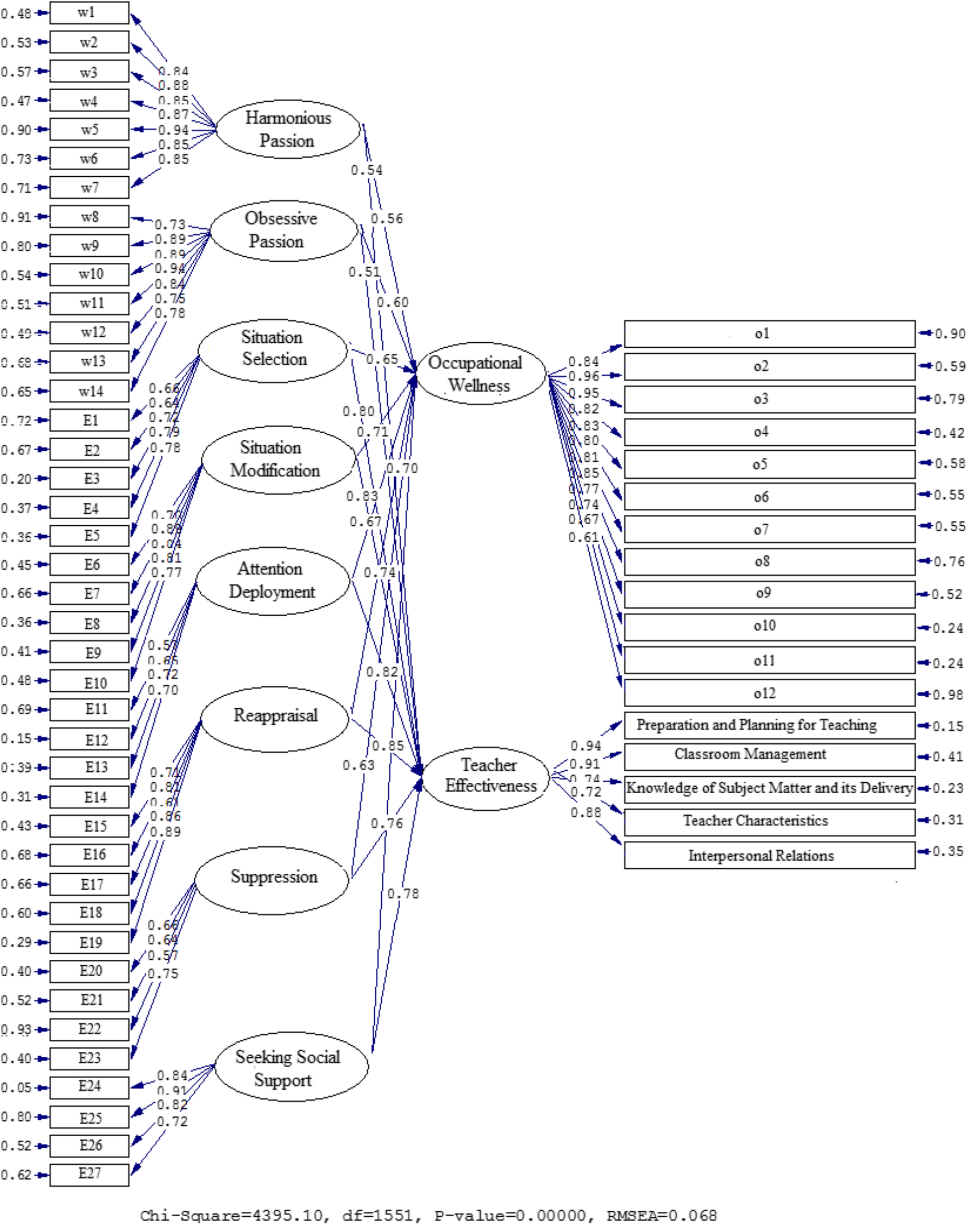
The Path Coefficient Values for the Interconnections between OW, TE, WP, and ER (Model 2)

**Fig. 4:**
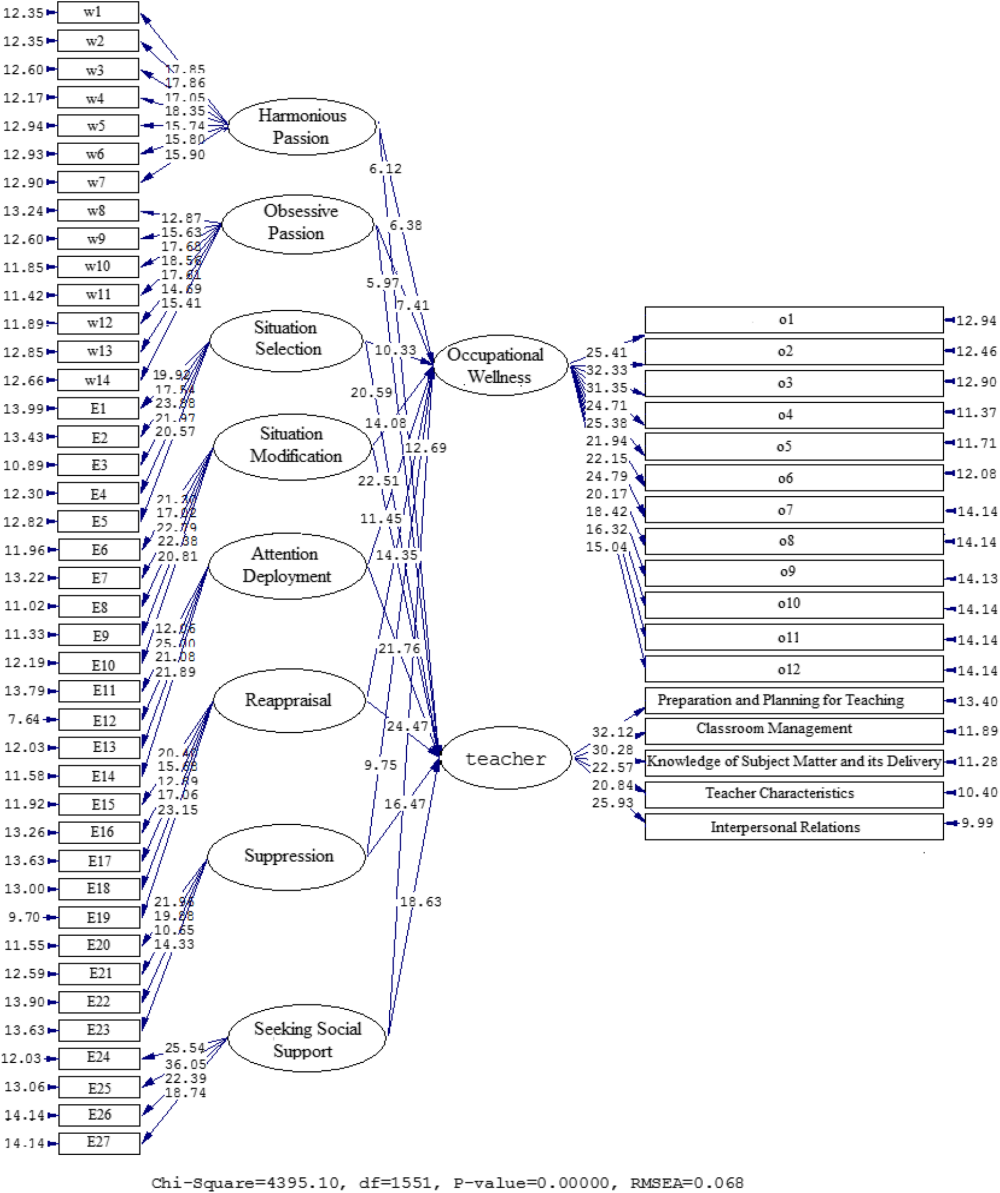
Path Coefficients with T Significance Values (Model 1)

Figures 3 and 4, together with Table 4, demonstrate the connections found between the WP and ER components as well as OW and TE. A correlation was discovered between OW and Harmonious Passion (β = 0.54, t = 6.12), Obsessive Passion (β = 0.51, t = 5.97), Situation Selection (β = 0.80, t = 20.59), Situation Modification (β = 0.71, t = 14.08), Attention Deployment (β = 0.67, t = 11.45), Reappraisal (β = 0.74, t = 14.35), Suppression (β = 0.63, t = 9.75), and Seeking Social Support (β = 0.70, t = 12.69). The relationships between TE and Harmonious Passion (β = 0.54, t = 6.12), Obsessive Passion (β = 0.51, t = 5.97), Situation Selection (β = 0.80, t = 20.59), Situation Modification (β = 0.83, t = 22.51), Attention Deployment (β = 0.82, t = 21.76), Reappraisal (β = 0.85, t = 24.47), Suppression (β = 0.76, t = 16.47), and Seeking Social Support (β = 0.78, t = 18.63) are also valid.

**Table 4:** A synopsis of the results of model 2

**Paths**			**Path coefficient**	**T Statistics**	**Test results**
Harmonious Passion	→	Occupational Wellness	0.56	6.38	Supported
Obsessive Passion	→	Occupational Wellness	0.60	7.41	Supported
Situation Selection	→	Occupational Wellness	0.65	10.33	Supported
Situation Modification	→	Occupational Wellness	0.71	14.08	Supported
Attention Deployment	→	Occupational Wellness	0.67	11.45	Supported
Reappraisal	→	Occupational Wellness	0.74	14.35	Supported
Suppression	→	Occupational Wellness	0.63	9.75	Supported
Seeking Social Support	→	Occupational Wellness	0.70	12.69	Supported
Harmonious Passion	→	Teacher Effectiveness	0.54	6.12	Supported
Obsessive Passion	→	Teacher Effectiveness	0.51	5.97	Supported
Situation Selection	→	Teacher Effectiveness	0.80	20.59	Supported
Situation Modification	→	Teacher Effectiveness	0.83	22.51	Supported
Attention Deployment	→	Teacher Effectiveness	0.82	21.76	Supported
Reappraisal	→	Teacher Effectiveness	0.85	24.47	Supported
Suppression	→	Teacher Effectiveness	0.76	16.47	Supported
Seeking Social Support	→	Teacher Effectiveness	0.78	18.63	Supported

Furthermore, according to [39], the chi-square is considered to be insignificant, and the ratio of chi-square to degrees of freedom ought to be less than three. It is also noted that the root means square error of approximation (RMSEA) should be less than 0.1. Additionally, a good match is indicated by the NFI where the cut value is larger than 0.90, the GFI where the cut value is higher than 0.90, and the CFI where the cut value is greater than 0.90 [39].

As can be shown in Table 5, Model 1 fit criteria are met by the chi-square/df ratio of 2.819, the RMSEA of 0.067, the GFI of 0.925, the NFI of 0.934, and the CFI of 0.959. Additionally, Table 5 provides a brief overview of the fact that every model fit index associated with Model 2 is suitable. Examples include the RMSEA (0.070), the GFI (0.945), the NFI (0.938), the CFI (0.952), and the chi-square/df ratio (2.908).

**Table 5:** Model fit indices

Fitting indexes	${\varvec{\upchi}}2$	$\mathbf{d}\mathbf{f}$	${\varvec{\upchi}}2/\mathbf{d}\mathbf{f}$	RMSEA	GFI	NFI	CFI
Cut value			<3	<0.1	>0.9	>0.9	>0.9
Model 1	696.39	247	2.819	0.067	0.925	0.934	0.956
Model 2	4395.10	1551	2.908	0.070	0.945	0.938	0.952

Moreover, this research used a Pearson product-moment correlation to examine the association between OW, TE, WP, and ER.

Table 6 displays the connections between OW, TE, WP, and ER that were determined to be significant. It was found that WP was associated with OW (*r* = 0.615) and TE (*r* = 0.602). The connections between ER and OW (*r* = 0.745) and TE (*r* = 0.841) were also confirmed. The details of these connections are illustrated in Table 6.

**Table 6:** The correlation coefficients between OW, TE, WP, and ER

	Occupational Wellness	Teacher Effectiveness	Work Passion	Emotion Regulation
Occupational Wellness	1.000			
Teacher Effectiveness	0.602**	1.000		
Work Passion	0.615**	0.556**	1.000	
Emotion Regulation	0.745**	0.841**	0.578**	1.000

Correlation is significant at the 0.01 level (2-tailed) **

Table 7 illustrates the positive and statistically significant link that exists between the OW, TE, WP, and ER subcomponents. OW and harmonious passion (*r* = 0.593), obsessive passion (*r*=0.624), Situation Selection (*r*=0.678), Situation Modification (*r* = 0.734), Attention Deployment (*r*=0.703), Reappraisal (*r*=0.760), Suppression (*r* = 0.655), and Seeking Social Support (*r* = 0.721), all supported this conclusion. Furthermore, it was established that the subsequent correlations between TE, WP, and ER subfactors were positive and statistically significant: harmonious passion (*r*= 0.560), obsessive passion (*r*=0.531), Situation Selection (*r*=0.824), Situation Modification (*r* = 0.856), Attention Deployment (*r*=0.841), Reappraisal (*r*=0.879), Suppression (*r*=0.780), and Seeking Social Support (*r* = 0.804).

**Table 7:** The correlation coefficients between the sub-components

	Occupational Wellness	Teacher Effectiveness	Harmonious Passion	Obsessive Passion	Situation Selection	Situation Modification	Attention Deployment	Reappraisal	Suppression	Seeking Social Support
Occupational Wellness	1.000									
Teacher Effectiveness	0.602**	1.000								
Harmonious Passion	0.593**	0.560**	1.000							
Obsessive Passion	0.624**	0.531**	0.556**	1.000						
Situation Selection	0.678**	0.824**	0.613**	0.721	1.000					
Situation Modification	0.734**	0.856**	0.625**	0.634**	0.542**	1.000				
Attention Deployment	0.703**	0.841**	0.705**	0.625**	0.617**	0.448**	1.000			
Reappraisal	0.760**	0.879**	0.671**	0.544**	0.635**	0.680**	0.708**	1.000		
Suppression	0.655**	0.780**	0.489**	0.628**	0.712**	0.651**	0.722**	0.634 **	1.000	
Seeking Social Support	0.721*	0.804**	0.553**	0.587**	0.746**	0.773**	0.678**	0.742 **	0.559 **	1.000

Correlation is significant at the 0.01 level (2-tailed) **

## Discussion

The overall theme of this research was to investigate the impact of EFL university teachers' WP and ER on their OW and TE. The results, particularly Model 1, indicate that WP and ER are important factors in predicting OW and TE. In accordance with the findings, notably Model 1, WP and ER are significant determinants in determining the likelihood of OW and TE that will occur. Self-determination, autonomy, enthusiasm, resilience, and persistence are all traits that may be developed via the cultivation of a powerful degree of protection and a skilled ability to manage emotions. Disregarding one's emotional balance and displaying maladaptive emotional intelligence, on the other hand, might have negative consequences. As a result, teachers in higher education need to use more ways that are contemplative and self-analytical to face the complex challenges and changes that are occurring in educational contexts [40]. It is of the utmost importance to improve teachers' understanding of the underlying principles that underlie the notions of WP, ER, OW, and TE, as well as the critical role that these competencies play in their job performance.

Firstly, it was revealed that enthusiasm for teaching and emotion management was an essential predictor of teacher OW (QR1: Are work passion and emotion regulation for EFL university teachers indicative of their occupational wellness?). This conclusion is corroborated by various researchers who have emphasized the relevance of teachers’ emotion control in classroom environments and its consequences on teacher welfare [41, 42]. It is plausible to suggest that instructors’ coping methods could enhance the association between emotional control and emotional health. For instance, teachers who apply more suitable emotional control tactics may suffer less fear from disordered learning situations and learners’ disobedience as they are adept at addressing stresses that arise in the educational setting. As a consequence, the perception of capability may make it possible for educators to achieve a higher level of psychological well-being, which in turn helps them to increase the likelihood that they will be satisfied with their job and the teaching they do in the classroom [42, 43]. On the other hand, when EFL instructors are unable to successfully manage their emotions, they are unable to successfully cope with the challenges that arise in the classroom; thus, they may consider their job to be emotionally draining [12, 43, 44]. In addition, teachers who can control their emotions can devise strategies that are suitable for their emotional state and establish a profound and pleasurable connection with their students. Consequently, teachers are more likely to experience satisfaction and joy in their work, and they are also more likely to boost their personal growth as they achieve strong emotional well-being throughout their careers. This conclusion is congruent with study findings of [45] which indicated that instructors’ effectiveness judgments were connected with their psychological and physical participation in instructional activities.

Secondly, it was established that teacher perception of effectiveness may strongly predict the psychological wellness of EFL instructors. This conclusion corresponds with the outcomes of a substantial body of literature indicating that high levels of teachers’ self-efficacy are connected with an elevated state of emotional wellness [46]. For instance, [47] observed that the self-worth of teachers was connected to their overall mental health. In other words, instructors with high self-efficacy reported greater degrees of good emotions and contentment and had a lower proportion of unfavorable sentiments. A possible reason could be that educators with a higher level of optimism (e.g., perceptions that they have a substantial effect on student’s development and learning) may be extremely motivated and exceedingly satisfied with their profession, which in turn may enhance their emotional health. This is corroborated by [48] who suggested that one’s intrinsic drive adds to their psychological wellness. Additionally, instructors’ enthusiastic mindset about their instruction could assist them in nurturing their teaching abilities and instructional efficacy by palliating their emotional burdens and problems. It may also be suggested that educators have a feeling of fulfillment and suffer less fatigue if they are equipped with stronger efficacy views and confidence in their skills to teach effectively and energetically engage their learners.

It may be suggested that educators’ substantial degree of competency and efficacy in their teaching abilities would lessen the levels of fear, dissatisfaction, and sadness. This explanation fits with the findings of several recent research that identified teacher self-efficacy as a negative indicator of disengagement and burnout [49]. From this approach, greater degrees of self-worth in teaching could be associated with increased work satisfaction and positive employment desires. The truth is, that teachers with a higher degree of instructional efficacy may grow confidence-boosting in employing methods for emotional regulation if they run into challenging and demanding situations; subsequently, they encounter fewer concerns in their position than instructors who have lower degrees of self-worth. As a consequence, teachers’ good feelings (i.e., reduced worry and further job satisfaction) might boost their psychological welfare as well as optimum functioning, pushing them to further devotion to instructional operations and work commitment. This is corroborated by [50] who illustrated that teachers’ contentment in educational settings is highly connected with their involvement levels. This research provides important insights on OW and TE with the mediator roles of WP and ER, and it has significant implications for policymakers, teacher educators, and other relevant stakeholders who are interested in understanding how to help language teachers in their professional duties.

## Conclusion

All in all, under the surface layer of teacher occupational wellness and effectiveness different factors are hidden. The study findings uncover that WP and ER are critical in determining the state of OW and TE in higher education. These results have significant implications for instructional administrators as a whole and the executive boards of higher education institutions specifically. The outcomes confirmed the significance of a teacher's WP and ER as strategic instruments for enhancing their OW and TE. Therefore, based on the results of this study, it is recommended that educators and strategists in higher education institutions focus on enhancing students' WP and ER by investing in the development of teachers' skills in this regard. This can be achieved by increasing teachers' knowledge through opportunities by providing them with training specifically tailored for teaching in higher education. The results of this research also provide evidence that emotional contagion fully mediates the transmission of a teacher's professional enthusiasm. This discovery indicates that a teacher's intense enthusiasm for their job may influence the ways they teach and act. Higher education planners must prioritize teachers' education. The results of this study indicate that the transmission of a teacher's WP and ER to OW and TE is more pronounced when the instructor has a Ph.D. degree. The results suggest that tactics in educational contributions and recruiters/hiring managers in higher education institutions should review faculty recruitment and selection policies. This will help them assign the most competent applicants with higher education and requirements, ensuring the delivery of high-quality education.

Higher education faculty may benefit from considering the instructional implications of this study's results. Higher education programs should take into account the need to teach students how to effectively use ER techniques based on contextual and psychological variables. A wide variety of tactics should be the focus of such training programs, with an emphasis on demonstrating when and how each one works. Training should also place more emphasis on how university teachers' personal qualities, as well as preferences, impact the efficacy of their ER tactics. This data also encourages academics to change their negative ER methods to more positive ones, which should help their self-efficacy and second language grit.

Professionals in the field of EFL education would do well to assist their fellow educators by providing them with guidance and training in the identification and management of stress, as well as in the development of healthy pedagogical attitudes and emotional regulation skills. Managers may do their part to create a warm and inviting classroom environment where EFL instructors feel comfortable sharing their true sentiments and where they can get the information, they need to accurately assess different teaching situations. If teachers have a more positive outlook on their work environment, they may be more motivated to find solutions to the emotional challenges they face in the classroom. The findings of this research highlight the need to support EFL professionals in developing their self-worth and knowledge since these factors are known to play a significant role in ensuring their psychological health while working in the EFL field. One way to make this happen is to provide teachers with more opportunities to acquire the knowledge they need to address any gaps in their pedagogical repertoire. Furthermore, to foster a more favorable perception of EFL instructors' ability as educators, supervisors or administrators should provide them additional power.

## Limitations and suggestions for future researchers

Some limitations are included in the results of this study: To begin, the research used a quantitative approach. Future research can utilize mixed-method approaches to examine the connections between various teacher-related concepts, such as ER, self-efficacy, L2 grit, work engagement, autonomy, critical thinking, job satisfaction, reflective teaching, self-regulation, and immunity, to gain a better grasp of the causal interactions among the variables in question. Secondly, as a point for future study, we did not investigate how participants' demographic factors impacted OW, TE, WP, and ER. Finally, individuals were selected via convenience sampling owing to practical limitations. Which is not accurately reflective. Consequently, findings from this research should be carefully understood and used broadly with caution. This research will concentrate on the ER methods used by EFL university teachers in the workplace. A trait method was used to evaluate EFL university teachers' ER. Moreover, ER methods in reaction to emotional events in the workplace were evaluated retrospectively, focusing on their frequency and intensity. Additionally, additional studies should be conducted to see whether ER affects their students' ER. Another idea is for academics to look at different types of educational settings, such as public and private language schools, to see how OW, TE, WP, and ER interact.

## Data Availability

The dataset of the present study is available upon request from the corresponding author.
